# Conjectures on some curious connections among social status, calorie restriction, hunger, fatness, and longevity

**DOI:** 10.1111/j.1749-6632.2012.06672.x

**Published:** 2012-07-26

**Authors:** Kathryn A Kaiser, Daniel L Smith, David B Allison

**Affiliations:** 1 Office of Energetics, School of Public Health, University of Alabama at Birmingham; 2 Nutrition Obesity Research Center, University of Alabama at Birmingham; 3 Department of Nutrition Sciences, School of Health Professions, University of Alabama at Birmingham

**Keywords:** hunger, fatness, caloric restriction, social status, longevity

## Abstract

Many animal and human studies show counterintuitive effects of environmental influences on energy balance and life span. Relatively low social and/or economic status seems to be associated with and produce greater adiposity, and reduced provision (e.g., caloric restriction) of food produces greater longevity. We suggest that a unifying factor may be perceptions of the environment as “energetically insecure” and inhospitable to reproduction, which may in turn provoke adiposity-increasing and longevity-extending mechanisms. We elaborate on two main aspects of resources (or the perceptions thereof) on body weight and longevity. We first discuss the effects of social dominance on body weight regulation in human and animal models. Second, we examine models of the interactions between caloric restriction, body composition, and longevity. Finally, we put forth a relational model of the influences of differing environmental cues on body composition and longevity.

## Introduction

Socioeconomic status is also related to the incidence and prevalence of obesity, such that the poor are disproportionately affected by obesity, regardless of race/ethnicity. Research is needed to further understand the impact of socioeconomic status on the development of obesity.^1^

A large body of research in animal models indicates that substantially reducing caloric intake while maintaining optimal nutrition results in significant increase in life span…^2^

Is perceived energetic uncertainty (in the face of a metabolizable energy surfeit) a key mediating variable in the causal chain leading to increased fat stores? Although the link between objective economic aspects of socioeconomic status (SES) and health outcomes is a familiar topic in the literature, body composition changes may also be significantly influenced by aspects of SES such as subjective social status (SSS). SSS is the self-evaluation of one's place within a community or group that is not necessarily proportional to immediate income or material resources. Despite the manifest implications in the contexts of obesity science and public health policy, the mechanisms of and the extent to which SSS is linked to obesity are unknown. We propose the novel hypothesis that it is the “socio” as much as or more than the “economic” in SES that increases obesity risk. That is, the self-perception of being low in a social hierarchy, independent of any specific, objective economic factors, may lead to physiological, cognitive, and behavioral changes that ultimately result in increased adiposity.

In this paper, we highlight and connect the current views on two aspects of energetics: (1) SES and obesity, and (2) caloric restriction and longevity. There is clear experimental and observational evidence for causative associations between SES and obesity as well as between caloric restriction and longevity. The implications of these two topics taken together are challenging to current paradigms. Both relationships have intriguing and complex research findings, and the exact mechanisms of these observed relationships remain elusive.

In brief, we explore the idea that animals (including humans) respond to perceived threats to their energetic security by switching life strategies to build and preserve energy stores to the extent that they can so as to buffer against true food scarcity that may occur later, and to extend life span, so as to breed more slowly or in better times. We begin with some long-standing observations and then offer conjectures about how these relationships operate in different populations of humans and animals. These observational and experimental studies include a variety of settings relating to general macroeconomics, food-specific economics, living environments, social perceptions, social hierarchies, economic uncertainty, hunger state, chronic hunger, food restriction, and energy uncertainty. We conclude with recommendations on future research directions.

## SES and obesity

### Is there a relationship?

In 1962, the classic Midtown Manhattan Study (which was originally conceived to examine social class and mental illness) revealed an unexpected inverse relationship between socioeconomic status (measured as a composite of the father's occupation and education) and body weight.^3^,^4^ This commentary from the 1962 paper describes the new observation and speculates excitably about some “seemingly obvious” implications:

The fact that obesity is 7 times more frequent in lower-class than in upper-class women has profound implications for theory and for therapy. For it means that whatever its genetic and biochemical determinants, obesity in man is susceptible to an extraordinary degree of control by social factors. It suggests that a broad-scale assault on the problem need not await further understanding of the physiological determinants of obesity. Such an assault might be carried out by a program of education and social control designed to reproduce certain critical influences to which society has already exposed its upper-class members.^4^

In their 1989 review of studies published after 1941, Sobal and Stunkard^5^ reported a strong, inverse relationship for women in developed societies, but inconsistent findings for men and children. For people in developing societies, Sobal and Stunkard found a strong positive relationship between SES and increasing body mass. More recent examinations of this relationship have focused on more specific aspects of the question. For example, in a 2005 study, the utility of SES measures in predicting significant weight increase was examined in 34 longitudinal studies performed in developed countries.^6^ The 1989 findings of Sobal and Stunkard were generally supported when education and occupation were used as predictors, but income was less consistently associated with obesity in this later review. Also, no relationship using any SES indicators was observed in studies with predominantly African (not African American) samples.^6^ In a 2008 review of cross-sectional studies of children reported between 1990 and 2005, a stronger inverse association was found between adiposity and parental education than between adiposity and parental occupation or income.^7^ Furthermore, in examining these more recent studies, Shrewsbury and Wardle found that SES was inversely associated with adiposity in 10 of the 18 studies in younger children (5–11 years), which supports the proposal that SES-related gradients in adiposity develop early in the life course.^7^ These results differed from what Sobal and Stunkard found for child studies in their 1989 review^5^ in that it appears that during the latter part of the 20th century, positive associations between SES indicators and obesity were rarely observed. This difference may be the result of increasing weight in all social classes or perhaps more severe obesity being increasingly prevalent in lower SES groups.

In a more recent examination of this question in children, the National Longitudinal Survey of Youth (NLSY; a 15-year cohort reported in 2006), excess body weight was inversely related to childhood SES (using mother's education as a proxy) and this disparity increased with age. Using the NHANES (National Health and Nutrition Examination Survey) to compare White, Black, and Mexican American children, Wang and Zhang found that for white adolescent boys and girls, there was a significant inverse association between SES and obesity in the NHANES III (1988–1994) sample.^8^ In contrast, Black girls with a high SES had a higher prevalence than did their low- and medium-SES counterparts in the 1988–1994 and 1999–2002 samples. For Mexican American children, the study sample was small and no consistent patterns were observed.^8^ Explanations for the observed racial disparities and causal mechanisms remain obscured by nonexperimental approaches.

### SES and obesity—is the relationship causal?

Thus far, the evidence for the SES-obesity link we have highlighted is based on strictly observational data, demonstrating correlation, but not necessarily causation. Other approaches are needed to assess causality and both quasi-experimental and experimental studies have recently been reported. One large randomized controlled trial examining living conditions and obesity was the Move to Opportunity (MTO) study.^9^ Between 1994 and 1998, 4498 women with children who were living in urban, high-poverty census tracts (≥40% of residents below the federal poverty level) were randomly assigned to one of three groups: one group received no vouchers, one received housing vouchers with no specific directions on where they might use them, and the third group were directed to use the housing vouchers they received to move to an area of low poverty (<10% of residents below the poverty level). At the 10- to 15-year follow-up, the group that moved to low-poverty areas had significantly lower percentages of women with body mass index (BMI) values above 35 and 40 and significantly lower glycated hemoglobin levels than did the control and nonspecific voucher groups. A follow-up analysis of the MTO study (Zhao, 2008; unpublished dissertation) examined several specific neighborhood factors of the locations the participants moved to, including food prices, availability of restaurants and food stores, availability of facilities for physical activity, crime, and population density. These factors had little impact on the intention-to-treat effects. This study supports the suggestion of Sobal and Stunkard in 1962 that perhaps the relative economic conditions of neighbors in the living environment are a “critical influence” on obesity.

In a recent analysis of two twin adoption cohort studies of over 2000 families, researchers sought to determine whether the association between rearing parent SES and adoptee BMI was statistically significant, even when controlling for the BMI of the rearing parents. In a form of “natural randomization,” both datasets suggested that shared genetic diathesis and direct environmental transmission contribute approximately equally to the association between rearing parent SES and offspring BMI.^10^ If the underlying mechanisms of these effects were understood, an intervention might be expected to reduce obesity at a level equivalent to the associated causal influence. In some cases, this might be a large effect.

One intriguing way in which causal inference about some aspects of the SES-obesity link could be achieved is through prospective studies using lottery and casino winners versus losers. These events are naturally occurring randomizations. Although such studies would address the single SES indicator (income) that is often least associated with weight, careful data collection and study design could yield new insights as to the effect of increased income on weight and other aspects of health. Randomized trials of income supplementation have been done, but without the examination of obesity or weight change as outcomes.^11^ Connor *et al.* suggested that if trials could be performed on contest winners or by randomizing recipients by use of unclaimed public funds, the results could inform policy deliberations on taxes, public benefits or entitlements, and minimum wage levels.^11^ Sudden changes in financial resources may or may not influence self-perceived social status.

### Effects of social perceptions on food intake and body composition

In humans, lower SSS is associated with higher BMI and waist-to-hip ratio to an even greater extent than is objective income.^12^ Differences in social status have also been shown to influence food intake patterns in animal studies. Some bases for this view include that subordinate status birds across many species (willow tit, great tit, greenfinch, chickadees, titmouse, nuthatch) carry greater fat reserves than do dominant status birds.^13^,^14^ In contrast to naturalistic observations in birds, controlled laboratory studies in primates provide further insight to socially subordinate effects. In nonhuman primates, socially subordinate females have demonstrated chronic psychological stress, have reduced glucocorticoid negative feedback, and have higher frequencies of anxiety-like behavior than do the socially dominant females.^15^ Twenty-four-hour intakes of both low- and high-fat diets were significantly greater in subordinates than in dominants, an effect that persisted whether standard monkey chow (13% of calories from fat) was present or absent. Additionally, feeding patterns were altered in subordinates: dominants restricted their food intake to daylight but subordinates continued to feed at night.^15^

### Social status and body composition in rodents

Perceptions of food availability may have effects in settings where social status is involved. Whereas the previous example in primates demonstrates a situation in which more energy consumed results in greater adiposity in subordinate females, the social stress associated with group housing of male rats has complex effects on body weight and body composition that depend on the hierarchical status of the rat within the group. Upon initial exposure to the social group dynamic in a visual burrow system (VBS), all animals lose weight comprising both lean and fat mass, with the subordinate males losing more weight (and more lean mass) than their dominant counterparts.^16^ This greater reduction in body weight is evident despite an equal or greater intake of food per gram of body weight;^16^ however, on a per animal basis, the food intake is often reduced in stress situations or subordinate animals. When removed from the VBS system and allowed to recover, both dominant and subordinate males gain weight rapidly, recovering the weight lost and reaching a significantly higher body weight, with no significant difference between the groups by approximately three weeks.^16^ Subsequent exposure to the social stress in the VBS produces similar weight loss patterns (subordinates lose more than do dominant animals), with subordinate males having a significantly greater body fat percentage after removal and recovery for three weeks, particularly in the visceral fat depots.^16^,^17^ Importantly, all animals in this experimental design are fed *ad libitum* (ad lib); thus, this is not an “economic” effect, but rather a social effect. It appears that the perception of social stress alters the physiologic response, thus improving metabolic efficiency and leading to greater body fat gains when the animal is removed from the socially stressful environment. Would such a response be expected in humans as we interact in multiple social groups at varying levels throughout our daily life and over the course of life? Additionally, how would an individual's perception of being subordinate or dominant alter these physiologic responses, particularly in an environment where exposures are intermittent and energetic availability is not limited?

### What if we all ate just a little bit less?

When mice are provided ad lib access to a palatable diet and limited exercise capacity, much like humans, they adopt a fairly sedentary lifestyle and overeat, which results in a positive energy balance and weight gain over time. To reduce this behavior, researchers sometimes limit the amount of food provided to control fed animals by ∼5% compared to what their ad lib counterparts would voluntarily eat.^18^ This is suggested to reduce excess weight gain, decrease variability among animals, and improve health. Somewhat unexpectedly, female mice in which intake was restricted by 5% of ad lib intake for one month of feeding exhibited a nonsignificant lowering of body weight but a significant reduction in lean mass and a significant increase in fat mass.^19^ That is, the animals were fatter even though they ate less. This phenotype was present across fat pad depots, including subcutaneous and visceral sites, reflecting a shift in energy assimilation and storage in the animals exposed to mild calorie restriction (CR) animals.^19^ Total energy expenditure and resting energy expenditure as measured by indirect calorimetry were both significantly lower in the 5% CR mice, although the diurnal pattern of energy expenditure remained intact with no significant difference in locomotor activity. Interestingly, feeding behavior was significantly different between groups despite this mild reduction, with 5% CR mice demonstrating gorging behavior immediately after food was provided, with no difference between groups for the rest of the dark cycle, but less intake during the day (light phase) in the 5% CR mice.^19^

One possible explanation for these results is that the small restriction resulted in daily depletion of food stores for the mice, which in turn induced daily hunger episodes that would not be present under ad lib conditions. Additionally, the gorging behavior could invoke a metabolic effect with the majority of energy intake coming in a single or limited number of meals, with little additional energy intake throughout the rest of the daily cycle. In line with these observations are the results of a study in which 41 recombinant inbred strains of mice were subjected to dietary restriction (fed 60% of ad lib intake) and then measured for body composition and longevity.^20^ Whereas CR normally reduces adiposity and increases longevity, life span extension was associated with fat preservation in these strains, with one strain significantly increasing fat mass in both sexes despite the 40% calorie reduction.^20^ How such a shift in the perception of food availability might translate to humans and body composition outcomes, in addition to more complex relationships of adiposity with health and longevity, merits further investigation.

A similar observation of social stress influencing energy intake (both caloric amount and timing of intake) with resulting body weight changes is also reported for nonhuman primates. Whereas rodents are found to eat less in stressed environments, excess consumption is proposed to be a major contributor to obesity development in humans.^15^ In one study, socially housed female macaques with a long-term, established dominance hierarchy were tested for three weeks during exposure to low-fat or high-fat diets. Consumption of either diet type was higher in subordinate animals than in dominant animals, during both day and nighttime measures across the three-week period. The increase in body weight over the three-week period was associated with the amount of intake, as might be expected, and was independent of social status. Thus, monkeys of lower social status consumed more calories with increased day and nighttime feeding than did their dominant counterparts and gained more weight.

In studies of dietary restriction, shifting female monkeys fed a high-fat diet ad lib to a restricted (30% fewer calories) low-fat diet resulted in a significant reduction in physical activity, which offset the energetic deficit sufficiently to prevent significant weight loss over a one-month period.^21^ A subsequent month of further restriction (60% fewer calories than ad lib) resulted in a further depression in physical activity, as well as a significant reduction in body weight, with no overall change in the percentage of body fat.^21^ Because these animals were singly housed, interactions related to social dominant or subordinate status were not obtained. Whether social hierarchy would influence this energetic compensation by altering physical behavior patterns remains to be explored. As a model for humans, nonhuman primate studies such as these could combine the social and energetic uncertainty elements to more comprehensively assess determinants for obesity-related outcomes pertinent to human social and environmental conditions.

### Perhaps it's the socio, not the economic

In contrast to food uncertainty or restriction in animals, in humans, Supplemental Nutrition Assistance Program (SNAP) participation (but not food insecurity) is associated with higher adult BMI in Massachusetts residents living in low-income neighborhoods.^22^ In 2005, a survey was done of 435 adult residents of low-income census tracts in Massachusetts. After adjustment for age, sex, sociodemographic characteristics, and food insecurity, both participation in the SNAP and participation in any federal nutrition program 12 months before the survey were each associated with an approximate 3.0 kg/m^2^ higher adult BMI. However, prolonged participation in the SNAP was associated with lower BMI. Persons who were eligible but did not participate had lower BMIs than did participants. This implies that perhaps short-term participation in the SNAP is associated with greater risks for obesity or perhaps that the psychosocial mechanisms associating food insecurity and obesity diminish over time in the SNAP.

### Economic uncertainty

In their study of the 1979 cohort of the Longitudinal Study of Youth, Smith *et al.* found that economic variability and uncertainty (as measured by probability of unemployment, number of income drops, volatility of income, and probability of being in poverty) were related to an increase in body fat.^23^ The authors asserted that body fat serves as an “insurance plan” against starvation, with greater risk requiring greater insurance. The economics of uncertainty and the relationship to food intake have been experimentally studied in animals.

Studies in mice indicate that two factors interact to determine the number of meals and meal size.^24^ These cost factors have been termed *approach cost* (procurement) and *unit cost* (consummatory). Results from varying schedules of approach and unit costs indicate that meal patterns in mice are sensitive to approach cost, whereas the total amount consumed is more sensitive to the unit cost. It was shown previously that when mice are forced to pay a price for food (e.g., to work in the form of bar presses or nose pokes), their daily intake is maintained well at lower prices but declines at higher prices, which is a relatively inelastic but classic consumer demand function.^24^ At relatively low costs (up to 25 responses), mice maintain body weight or slow growth that is comparable to no-cost conditions. The feeding patterns under these conditions show that mice “graze” during the first part of the night with undefined meals, consistent with a period of almost continuous home cage activity, but show more defined, small meals during the latter part of the night, eating very little by day.^25^

### Economic disparity and obesity

The Gini coefficient, which was developed by Italian statistician and sociologist Corrado Gini, was proposed in 1912 as a measure of the inequality of income distribution.^26^ A Gini value of 0 indicates a perfectly equal distribution of income, and a value of 1 indicates maximal inequality in which one person in the country has all the income. In analyses of 23 high-income and 10 middle-income countries with less economic dispersion, Due *et al*. (2009) found that economic inequality as measured by the Gini coefficient was more important in explaining both level of and inequality in overweight among adolescents than was absolute economic level.^27^ These findings further suggest that inequality as disparity in wealth promotes weight gain.

### Hungry for money and other valued resources

Some researchers assert that the desire for money is an evolutionary extension of our innate desire for food. Briers *et al.* conducted three studies showing the reciprocal association between the incentive value of food and money.^28^ In study 1, hungry participants were less likely than were satiated participants to donate money to a charity. In study 2, participants in a room with an olfactory food cue, which is known to increase the desire to eat, offered less money in a “give-some” game than did participants in a room free of food smells. In study 3, the participants’ desire for money was associated with an increase in the amount of candies they ate in a subsequent taste test, but only among participants who were not restricting their food intake in order to manage their weight. Perhaps in present-day societies, the attraction to money is so powerful that people who, relatively speaking, fail in their quest for increased financial resources become frustrated. Because financial and caloric resources are valued as exchangeable, people might tend to appease their desire for money by consuming more calories than is necessary to sustain body weight.^28^

Examining effects of hunger on other resources of high value, Pettijohn *et al.* performed a field test of the *environmental security hypothesis*.^29^ This hypothesis is a context-dependent theory of attraction and preferences that draws on evolutionary theory and ecology. Researchers examined the effects of a hunger state on perceived attractiveness of idealized romantic partners.^30^ The results indicated that hungry male participants preferred ideal romantic partners who were relatively heavier (>120 lbs) and satiated males preferred ideal partners who were relatively lighter (<120 lbs), to a statistically significant degree. In assessing the influence of perceived maturity of idealized partners, hungry male participants preferred those who were slightly older than themselves and full males preferred ideal partners who were slightly younger.^30^ These findings together suggest a reliable influence of the evaluation of one's personal resources in a short-term context on perceptions of both food intake drive and mate preferences.

Hence, the economics of perceived food availability and hunger states seem related to food-seeking behavior as well as individual fat-storage strategies that are beyond conscious control. Next, we examine the effects of limited food (caloric restriction) on another important aspect of evolutionary fitness: longevity.

## Caloric restriction and longevity

Calorie restriction remains the most highly researched, nongenetic intervention to improve health and increase life span in research organisms ranging from single-celled yeasts to nonhuman primate models.^31^,^32^ These health and longevity benefits are proportional to the amount of restriction up to the point of malnutrition^33^ and are generally independent of the macronutrient content being restricted (fat versus carbohydrates versus protein). The data, considered as a whole, suggest a clear, causal relationship between energy provision and mortality rate, as well as senescence and metabolic-related disease.

The mechanisms of action for these observations remain unclear. One consistent phenotype of CR across animal models is the reduction of body weight and particularly body fat.^32^ In longevity studies using rodents, many ad lib-fed laboratory rodents develop age-associated obesity, even when fed a presumably healthy, low-fat diet. When adding a high-fat diet, most rodents exhibit a diet-induced obesity response similar to what is expected of humans who over-consume a calorie-rich, high-fat diet. With the switch from an ad lib diet to a restricted feeding paradigm, CR induces a rapid and sustained weight loss associated with the caloric deficit. This “negative energy balance” phase is followed by the establishment of a new equilibrium in which the reduced body weights are matched to the energy provision, i.e., relative energy balance. Therefore, the body weight and body composition changes associated with CR are more long term in nature than is the temporary energy deficit that is experienced during the initiation of CR. Thus, one could posit that the lower body weight or fat induced by CR partially mediates the effect of CR on life span. We tested this in a large sample of Wistar rats (*N*= 1200).^34^ The relative contribution of body weight to the CR effect was approximately 11%, thus supporting this hypothesis of partial mediation by low body weight. In a related study, we tested the influence of intentional weight loss by CR on mortality rates in outbred rats after the establishment of obesity. CR resulted in increased longevity compared with that in animals that remained obese, which suggests that weight loss can increase longevity even after the onset of obesity.^33^

In addition to the rodent model, two long-term, randomized CR studies in nonhuman primates are providing the first evidence that age-related mortality may be significantly lower in CR animals, concomitant with significantly reduced metabolic-related disease.^35^ Whether similar disease prevention and maximal longevity extension will be present in the CR monkeys is not yet established, but additional interim results for mortality are expected in the near term (1–3 years).

### CR and longevity: is perceived energetic uncertainty (independent of immediate energy intake) a mechanism of causation?

One aspect of the CR paradigm is feeding less than would be voluntarily consumed under similar but ad lib conditions. This results in animals that are both acutely and chronically hungry,^36^,^37^ which induces a gorging behavior by animals that are normally restricted once food is presented. Whereas ad lib fed animals have constant access to adequate food supplies, CR animals will often consume the majority of their daily food allotment shortly after it is given, resulting in extended periods of fasting during which food is neither available nor perceived by the senses. This means that not only are CR mice energetically restricted, but a host of neuroendocrine signals that coordinate energy intake and expenditure are presumably altered for most of the animal's life span. Because this may be perceived as a stressful situation, it raises the possibility that a small amount of something that is harmful in large doses may in fact be beneficial, i.e., the idea of hormesis.^38^–^40^ The idea of hormesis is often discussed in toxicology, where complex J- or inverted U-shaped curves—as opposed to a monotonic response—are observed with a dose-response pattern (i.e., just the right amount is beneficial, whereas either too little or too much is harmful). Whereas homeostasis is the process by which bodily functions are maintained, allostasis is the process by which bodily functions change in response to environmental challenges.^41^ It is plausible that the perception of energetic uncertainty is a hormetic signal leading to allostatic adaptive responding that both increases fat deposition when sufficient metabolizable energy is available and leads to increased life span if conditions are otherwise permissive.

Regarding CR and hunger, it seems reasonable that organisms must judge the nutrient and energetic state of their environment on the basis of specific cues. If, as in the case of CR, those cues are altered owing to impaired access to food supplies the majority of the time, might this elicit a protective response to promote the maintenance and preservation of the organism until a more favorable nutritional environment is encountered? Although one might expect these cues to be external from the environment, it is possible that internal cues like fat stores might also be coordinated in the response to balance the need versus availability equation of energy perception. Related to this idea, visceral adipose tissue is associated with increased morbidity and mortality and is proposed to play a role in multiple metabolic-related diseases. Importantly, visceral adipose depots are sensitive to energetic needs and are reduced in negative energy balance, that is, the CR state. By surgically removing part of the visceral fat in rats, significant health and longevity benefits have been achieved independent of CR, which suggests a role for fat and particularly visceral adipose tissue in mediating health and longevity.^42^ Another possible interpretation would suggest that visceral adipose tissue reduction by mechanical means removes a signal of surplus energy stores, which results in a perceived energetically “lean” time and contributes to the health and longevity benefit. Whether such a signal exists and is directly related to the specific fat depots themselves is unknown.

Perception of nutrient availability has also been studied in worms and flies in relationship to survival and mortality kinetics. *Drosophila melanogaster* has been used extensively in nutrition and aging research partly because of its relatively short life span and the ease with which researchers can produce and modify diets of known composition. Although it was previously known that CR increases life span in flies, recent studies have shown that mortality is acutely sensitive to nutrient availability.^43^ This was modeled by switching flies from dietary restriction (DR) to ad lib feeding (or vice versa—ad lib to DR), which meant switching them from being housed with standard, rich media access (ad lib) to a dilute media composition. Within two days of switching the flies to the opposite nutrient state, mortality rates paralleled those of animals continuously exposed to the nutrient condition.^43^ Although it is possible that dietary factors and intake amounts could produce a biological caloric effect resulting in survival modulations, the acute response suggests that additional factors such as nutrient or environmental perception were also involved beyond the proposed mechanisms of more long-term aging phenotypes such as accumulated cellular damage.^43^ This idea is further supported by results showing that the extended life span of DR flies could be shortened by sensing (e.g., smelling) live yeast that were present but not available for consumption.^44^ Importantly, the perception of live yeast did not shorten life span of ad lib fed flies, which suggests that the effect was not generally negative (like a toxic effect), but rather unique to the DR response. Further studies showed that disruption of olfactory receptor modulator (Or83b) alone was sufficient to increase life span and induce a number of stress-resistant phenotypes, thus supporting the role of perception in mediating multiple aspects of the DR related response.^45^ In agreement with other work in *C. elegans*,^46^,^47^ these studies demonstrate a critical role of sensory perception in the longevity response to DR and point to interactions and modulation of perception on complex phenotypes such as life span.

The hunger aspect of hormesis is further supported by work using intermittent feeding (IF) or every other day (EOD) feeding in rodents. Rather than daily restriction, the EOD or IF models permit ad lib feeding, but only for specific periods of time followed by complete fasting (e.g., one-day fed ad lib, one-day fasted, one-day fed ad lib, etc.). Because food intake during the ad lib period may not fully compensate for the fasting period, EOD feeding may result in mild energy restriction. Even in rodent strains that do not lose weight with EOD feeding, life span is increased when the protocol is started at young age.^48^,^49^ Comparing strains of rodents for the body weight and longevity response, it appears that life span is not fully predicted from changes in body weight. This suggests that other factors, possibly daily hunger, may be contributing to the benefit.

### If the perception of restriction were beneficial, would repeated bouts of weight loss be beneficial?

As mentioned previously, CR is known to have positive effects on health and longevity, whereas obesity and excess adiposity are detrimental. What would happen if all individuals who were overweight or obese lost weight to a normal body size? Although this is not likely to occur, most of those who successfully lost the weight would regain the amount within a few years.^50^ If, as is commonly believed, excess weight and adiposity are harmful, would it be beneficial (or harmful or have no effect) to go from overweight to normal and back again? Yo-yo dieting and weight loss is certainly observed in humans, and although not normally encountered in laboratory nutrition studies, this question of the potential health consequences with weight cycling from repeated bouts of weight loss and regain is beginning to be addressed. A study is currently underway at the University of Alabama at Birmingham in which high-fat diet feeding (similar to the Western diet macronutrient composition) is utilized to establish an overweight/obese cohort of mice. The mice are subsequently randomized to interventions in which food intake is continued ad lib, restricted sufficiently to achieve a normal body weight, or food intake is restricted and then refed similar to a yo-yo dieting experience. With the CR phase, animals clearly lose weight, and when released to ad lib feeding, usually return to approximately their pre–weight loss size or greater. Thus, not only is this study a model of CR for intentional weight loss (and regain with yo-yo dieting), these animals experience varied amounts and time periods of imposed hunger by CR. It may be that treating overweight may be beneficial to do at regular intervals, even with modest results, similar to the way that oral health benefits from regular plaque removal (i.e., a “dental model of obesity treatment”). Although a person may not be able or motivated to prevent the buildup of plaque at the gum line (which is not viewed as a treatment failure), frequent plaque removal efforts by dental hygienists reduce the long-term, health-damaging effects.

### Other evidence for the stress/hormesis hypothesis

Stress comes in a variety of forms and, as proposed in this work, does not necessarily require a specific physical or biological insult but rather may arise from the organism's perception of its relative state or environment. Multiple stressors have been discussed in association with life span modulation, including environmental temperature and osmotic stress.^39^,^51^ The extent to which these survival-altering interventions operate through overlapping pathways and mechanisms that are critical for life span extension and that are mediated by a stress response remains to be demonstrated.

### What might this imply about calorie restriction mimetics?

Considering that the perception of nutrients through hunger or other pathways may mediate the effect of CR, might it be possible to illicit a similar physiologic response without requiring actual CR? This concept of mimicking the benefits of CR without actual caloric reduction was first proposed by Ingram *et al.*, in part, on the basis of their work with multiple compounds that would interact with metabolic pathways implicated in CR.^52^–^54^ Although multiple compounds have been identified that induce a similar physiologic and transcriptional response to CR, few compounds have produced increased longevity in animal studies. Two of the earliest proposed CR mimetics were metformin and 2-deoxyglucose (2DG). Multiple rodent longevity studies are reported for metformin supplementation,^55^–^57^ and there is one recent longevity report for 2DG.^58^ One important aspect of these CR mimetics is how comprehensively the mimetic recapitulates the total CR response. Regarding the hunger associated with CR, there is little reason to suspect that metformin would elicit a hunger response. Thus, any hunger-associated hormetic response would be lacking. Similarly, with 2DG, no increase in food intake was observed,^58^ which suggests a lack of overt hunger. The clear toxic effects of high 2DG make dosing a challenge; therefore, what dose of either compound, if any, could elicit a hunger response similar to CR is unclear. Whether additional CR mimetics should require demonstrated or reported hunger as part of the response is an open question. Current collaborations with the Interventions Testing Program of the National Institute on Aging testing an α-glucosidase inhibitor (acarbose) may provide useful information on a hunger-hormetic mechanism of action. Acarbose is expected to induce a hunger response in addition to metabolic and physiologic improvements. A related approach is being considered using ghrelin, a gut-derived peptide associated with feeding and hunger that could induce a chronic, artificial hunger state despite access to energetic requirements sufficient to meet the metabolic demands of the organism.

### Implications if these hypotheses are true

**Figure 1:**
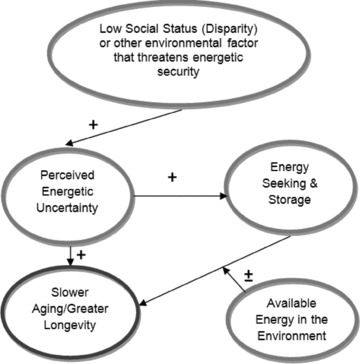
Hypothesized model of social and environmental influences on energy balance that affect aging.

We offer the diagram on the previous page (Fig. 1) as an integrated model of the observations and effects we have discussed. Using this model, future efforts to identify leverage points that affect perceptions of energetic uncertainty may be as important as or more important than identifying specific economic leverage points. Furthermore, environmental manipulations that lead to the perception of restricted food availability may have paradoxical effects. On a physiological level beyond conscious perceptions, some CR mimetics may need to work downstream of hunger signals to be effective. Because of the apparent cascade of processes in CR, even discounting side-effects, losing weight with an anorexigen may be less beneficial than losing an equivalent amount of weight with CR. Some CR-like interventions that do not actually reduce total energy intake may also lead to prolonged life. Our proposed model provides a testable framework in which future investigations might illuminate mechanistic pathways.

## Conclusions

There is much to be understood if we are to reduce the undesirable human and economic costs that accompany unhealthy aging, reduced life span and obesity-related chronic illnesses. A large body of literature provides clues to the importance of how social and other environmental cues may operate in these contexts, based on evolutionary strategies that govern the protection of reproductive ability. We have provided a novel framework connecting social status, adiposity, and longevity that offers specific testable predictions and look forward to future research testing those predictions.
